# Inverse Propensity Score-Weighted Analysis of Entecavir and Tenofovir Disoproxil Fumarate in Patients with Chronic Hepatitis B: A Large-Scale Multicenter Study

**DOI:** 10.3390/cancers15112936

**Published:** 2023-05-26

**Authors:** Jihye Kim, Moon Haeng Hur, Seung Up Kim, Jin-Wook Kim, Dong Hyun Sinn, Hyun Woong Lee, Moon Young Kim, Jae Youn Cheong, Yong Jin Jung, Han Ah Lee, Young-Joo Jin, Jun Sik Yoon, Sung-Jae Park, Chang Hun Lee, In Hee Kim, June Sung Lee, Young Youn Cho, Hyung Joon Kim, Soo Young Park, Yeon Seok Seo, Hyunwoo Oh, Dae Won Jun, Mi Na Kim, Young Chang, Jae Young Jang, Sang Youn Hwang, Yoon Jun Kim

**Affiliations:** 1 Department of Internal Medicine, Seoul National University College of Medicine, Seoul National University Bundang Hospital, Seongnam 13620, Republic of Korea; 2 Department of Internal Medicine and Liver Research Institute, Seoul National University College of Medicine, Seoul 03080, Republic of Korea; 3 Department of Internal Medicine and Yonsei Liver Center, Severance Hospital, Yonsei University College of Medicine, Seoul 03722, Republic of Korea; 4 Department of Internal Medicine, Samsung Medical Center, Sungkyunkwan University School of Medicine, Seoul 06351, Republic of Korea; 5 Department of Internal Medicine, Gangnam Severance Hospital, Yonsei University College of Medicine, Seoul 06273, Republic of Korea; 6 Department of Internal Medicine, Yonsei University Wonju College of Medicine, Wonju 26426, Republic of Korea; 7 Department of Gastroenterology, Ajou University School of Medicine, Suwon 16499, Republic of Korea; 8 Department of Internal Medicine, Seoul Metropolitan Government Seoul National University Boramae Medical Center, Seoul 07061, Republic of Korea; 9 Department of Internal Medicine, Ewha Womans University College of Medicine, Seoul 07985, Republic of Korea; 10 Department of Internal Medicine, Inha University Hospital, Inha University School of Medicine, Incheon 22332, Republic of Korea; 11 Department of Gastroenterology and Hepatology, Inje University Busan Paik Hospital, Busan 47392, Republic of Korea; 12 Department of Internal Medicine, Jeonbuk National University Hospital, Jeonju 54907, Republic of Korea; 13 Department of Internal Medicine, Inje University Ilsan Paik Hospital, Goyang 10380, Republic of Korea; 14 Department of Internal Medicine, Chung-Ang University College of Medicine, Seoul 06973, Republic of Korea; 15 Department of Internal Medicine, School of Medicine, Kyungpook National University Hospital, Kyungpook National University, Daegu 41944, Republic of Korea; 16 Department of Internal Medicine, Korea University Anam Hospital, Korea University College of Medicine, Seoul 02841, Republic of Korea; 17 Department of Internal Medicine, Uijeongbu Eulji Medical Center, Eulji University School of Medicine, Uijeongbu 11759, Republic of Korea; 18 Department of Internal Medicine, Hanyang University Hospital, Hanyang University College of Medicine, Seoul 04763, Republic of Korea; 19 Department of Internal Medicine, CHA Bundang Medical Center, CHA University, Seongnam 13496, Republic of Korea; 20 Department of Internal Medicine, Soonchunhyang University College of Medicine Seoul Hospital, Seoul 04401, Republic of Korea; 21 Department of Internal Medicine and Gastrointestinal Cancer Center, Dongnam Institute of Radiological & Medical Sciences, Busan 46033, Republic of Korea

**Keywords:** entecavir, tenofovir, liver cancer, extrahepatic malignancy, virologic response

## Abstract

**Simple Summary:**

It is still controversial whether tenofovir disoproxil fumarate (TDF) has a lower risk of hepatocellular carcinoma (HCC) development than entecavir (ETV) in patients with chronic hepatitis B (CHB). Furthermore, other antiviral treatment-related outcomes need to be evaluated between the two antivirals to provide essential information for clinical practice. Using a multicenter cohort of 4210 CHB patients, we demonstrated that ETV and TDF are comparable in terms of HCC development as well as mortality, incidence of liver-related outcome, extrahepatic malignancy or new decompensation events, and seroconversion rates. Patients treated with TDF experienced more side effects than those treated with ETV. The results of this study can be applied to the development of a personalized antiviral treatment strategy for CHB patients.

**Abstract:**

Tenofovir disoproxil fumarate (TDF) is reportedly superior or at least comparable to entecavir (ETV) in preventing hepatocellular carcinoma (HCC) among chronic hepatitis B (CHB) patients; however, it remains controversial. This study aimed to conduct comprehensive comparisons between the two antivirals. CHB patients initially treated with ETV or TDF between 2012 and 2015 at 20 referral centers in Korea were included. The primary outcome was the cumulative incidence of HCC. The secondary outcomes included death or liver transplantation, liver-related outcome, extrahepatic malignancy, development of cirrhosis, decompensation events, complete virologic response (CVR), seroconversion rate, and safety. Baseline characteristics were balanced using the inverse probability of treatment weighting (IPTW). Overall, 4210 patients were enrolled: 1019 received ETV and 3191 received TDF. During the median follow-ups of 5.6 and 5.5 years, 86 and 232 cases of HCC were confirmed in the ETV and TDF groups, respectively. There was no difference in HCC incidence between the groups both before (*p* = 0.36) and after IPTW was applied (*p* = 0.81). Although the incidence of extrahepatic malignancy was significantly higher in the ETV group than in the TDF group before weighting (*p* = 0.02), no difference was confirmed after IPTW (*p* = 0.29). The cumulative incidence rates of death or liver transplantation, liver-related outcome, new cirrhosis development, and decompensation events were also comparable in the crude population (*p* = 0.24–0.91) and in the IPTW-adjusted population (*p* = 0.39–0.80). Both groups exhibited similar rates of CVR (ETV vs. TDF: 95.1% vs. 95.8%, *p* = 0.38), and negative conversion of hepatitis B e antigen (41.6% vs. 37.2%, *p* = 0.09) or surface antigen (2.8% vs. 1.9%, *p* = 0.10). Compared to the ETV group, more patients in the TDF group changed initial antivirals due to side effects, including decreased kidney function (n = 17), hypophosphatemia (n = 20), and osteoporosis (n = 18). In this large-scale multicenter study, ETV and TDF demonstrated comparable effectiveness across a broad range of outcomes in patients with treatment-naïve CHB during similar follow-up periods.

## 1. Introduction

Hepatitis B virus (HBV) infection affects approximately 300 million people worldwide, and chronic hepatitis B (CHB) is a leading cause of liver diseases in East Asia [1,2]. CHB can lead to serious complications such as liver cirrhosis and hepatocellular carcinoma (HCC). To reduce the risk of HCC in patients with CHB, both entecavir (ETV) and tenofovir disoproxil fumarate (TDF) have been widely used due to their potent viral suppressive efficacy and high genetic barriers to drug resistance. These two nucleos(t)ide-analogues (NAs) are currently recommended as the first-line antiviral drugs in CHB patients, along with tenofovir alafenamide (TAF), which has been introduced recently [3,4,5].

Despite the extensive efforts to reveal the superiority between ETV and TDF, it remains controversial whether TDF is superior or comparable to ETV for the prevention of HCC among CHB patients. Some studies showed that TDF is associated with a significantly lower risk of HCC compared to ETV [6,7,8,9,10,11,12], while others demonstrated that there was no remarkable difference between the two antivirals [13,14,15,16,17,18,19,20,21]. There has been no study confirming the superiority of ETV. Controversy on this issue has persisted since randomized controlled trials are practically unfeasible, considering the low incidence of HCC in CHB patients and the long average duration to detect HCC from the initiation of NA treatment. Moreover, it was suggested that the earlier introduction of ETV compared to TDF could have resulted in the false superiority of TDF by including more patients with high risks of HCC in the ETV group [14,15,16].

However, not only the incidence of HCC, but also other antiviral treatment-related outcomes should be carefully examined. Although the development of HCC can greatly impact the prognosis of each patient, overall HCC incidence is not high, especially in low-risk groups (e.g., young and female CHB patients without liver cirrhosis) [22]. In a recent study which estimated an individualized risk of HCC using an artificial intelligence model, CHB patients classified as a minimal-risk group exhibited less than 1% risk of HCC during 8 years [22]. Therefore, antiviral agents should be selected for those with low risks of HCC based on multiple factors, including viral suppressive efficacy and side effects.

Based on this background, we aimed to conduct comprehensive analyses to provide a wide range of data on ETV and TDF, the two most widely used antivirals. Using a large-scale multicenter cohort, in addition to the incidence of HCC, various outcomes considered essential in clinical practice, such as mortality, liver cirrhosis development in initially non-cirrhotic patients, prevention of decompensation events among cirrhotic patients, effectiveness of viral suppression, and rate of drug cessation or switch, were assessed.

## 2. Materials and Methods

### 2.1. Patients

Between December 2012 and August 2015, treatment-naïve CHB patients initially treated with either ETV or TDF were enrolled from 20 referral centers in South Korea. Because ETV and TDF have been used in Korea since 2007 and 2012, respectively, patients who initiated antiviral therapy after 1 December 2012 were enrolled so that the two groups would have comparable follow-up durations. Antiviral treatment was initiated according to the Korean Association for the Study of the Liver (KASL) guidelines [5]. Liver cirrhosis was confirmed either histologically or clinically (nodular liver margin with splenomegaly on abdominal imaging, presence of ascites, varices, or hepatic encephalopathy, and/or platelet counts less than 150,000/mm^3^) [23].

Exclusion criteria were as follows: (i) co-infection with hepatitis C virus, hepatitis D virus, hepatitis E virus, or human immunodeficiency virus; (ii) patients with other chronic liver diseases (autoimmune hepatitis, hemochromatosis, Wilson’s disease, primary biliary cholangitis, or alpha-1-antitrypsin deficiency); (iii) history of malignancy or organ transplantation; (iv) previous antiviral treatment with NA or interferon; (v) prophylactic antiviral therapy with ETV or TDF; (vi) any malignancy including HCC or liver transplantation (LT) within 1 year from the antiviral treatment; (vii) follow-up duration less than 1 year or cessation of NA treatment within 1 year; (viii) missing HBV DNA values or HBV DNA less than 2000 IU/mL; and (ix) warfarin users. The institutional review board of each hospital approved this study, and the consent of study participants was waived due to the retrospective nature of the study.

### 2.2. Variables

Data on baseline variables at the initiation of antiviral treatment were collected: age, sex, body mass index, comorbidities (diabetes mellitus and hypertension), presence of (decompensated) cirrhosis, hepatitis B e antigen (HBeAg) positivity, platelet count, serum levels of albumin, total bilirubin, aspartate aminotransferase (AST), alanine aminotransferase (ALT), prothrombin time, alpha fetoprotein (AFP), and HBV DNA. Duration between the index date and the event of interest was measured. Survival data were retrieved from the medical records of each hospital or the national database provided by the Ministry of the Interior and Safety of Korea.

### 2.3. Primary Outcome

The primary outcome was diagnosis of HCC, which was confirmed by either liver biopsy or image findings [24,25]. Patients received regular HCC surveillance using abdominal ultrasonography and serum AFP at least every 6 months from the index date (the date of antiviral treatment initiation) until the diagnosis of HCC, death, or last follow-up. In cases of inconclusive ultrasonographic findings, contrast-enhanced computed tomography and/or magnetic resonance imaging were also utilized.

### 2.4. Secondary Outcomes

A total of 10 secondary outcomes were evaluated: (i) Incidence of death from any cause or LT was compared. (ii) Incidence of liver-related outcome (LRO), defined as HCC development, LT, or liver-related death, and (iii) any extrahepatic malignancy (EHM) was estimated. (iv) Development of liver cirrhosis in initially non-cirrhotic patients and (v) occurrence of new decompensation events (variceal bleeding, hepatic encephalopathy, or ascites) among compensated cirrhosis patients were compared between the two treatment groups. (vi) Adherence rate, defined as a total prescription duration over a follow-up duration (i.e., from the first visit to the last visit), was calculated. The proportions of patients who achieved (vii) complete virologic response (CVR; HBV DNA level below 20 IU/mL) as well as (viii) HBeAg or (ix) hepatitis B virus surface antigen (HBsAg) negative conversion were evaluated. Lastly, we compared the (x) rate and cause of drug modification, including side effects. TDF was associated with a higher risk of adverse events, such as renal function impairment and osteoporosis, than ETV [26,27]. Hypophosphatemia was defined as a serum phosphorus concentration of less than 2.5 mg/dL, and renal insufficiency was defined as an increase in creatinine level of ≥0.3 mg/dL or a ≥1.5-fold increase from the baseline creatinine level [28,29]. Osteoporosis was diagnosed when a T-score was below −2.5 [30].

### 2.5. Statistical Analysis

Chi-squared and independent t-tests were used to compare categorical and continuous variables, respectively. To balance the baseline characteristics between the two groups, inverse probability of treatment weighting (IPTW) was employed [31]. Patients who changed the type of NA during follow-up were censored at the time of the modification. Several outcomes including the incidence of HCC, LRO, and EHM were calculated using the Kaplan–Meier method and compared between the two groups using the log-rank test. Subgroup analyses were performed to evaluate the impact of HBeAg or cirrhosis on the development of HCC. The Cox proportional hazard model was applied to identify the risk factors of HCC, and hazard ratio (HR) with 95% confidence interval (CI) was derived. All statistical analyses were performed using R (version 4.0.4; R Foundation for Statistical Computing, Vienna, Austria). *p* values from two-tailed tests with a level of less than 0.05 were considered statistically significant.

## 3. Results

### 3.1. Baseline Characteristics

**Figure 1:**
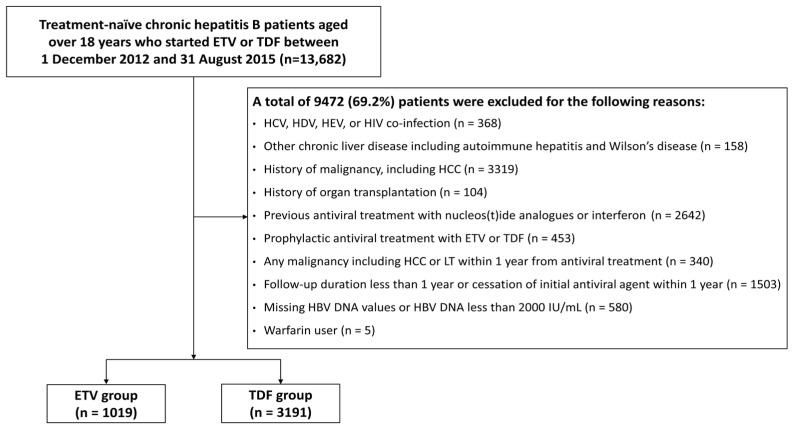
Patient flow diagram. ETV, entecavir; HBV, hepatitis B virus; HCC, hepatocellular carcinoma; HCV, hepatitis C virus; HDV, hepatitis D virus; HEV, hepatitis E virus; HIV, human immunodeficiency virus; LT, liver transplantation; TDF, tenofovir disoproxil fumarate.

This multicenter study enrolled a total of 4210 treatment-naïve patients with CHB, 1019 of whom were treated with ETV (the ETV group), and the others (n = 3191) received TDF (the TDF group; Figure 1). The baseline characteristics of the two treatment groups are summarized in Table 1. The ETV group included older patients with a higher proportion of comorbidities than the TDF group. Platelet count and serum levels of AST, ALT, AFP, and HBV DNA were measured lower in the ETV group compared to the TDF group. However, all variables were well balanced after IPTW was applied.

### 3.2. HCC Development

**Figure 2:**
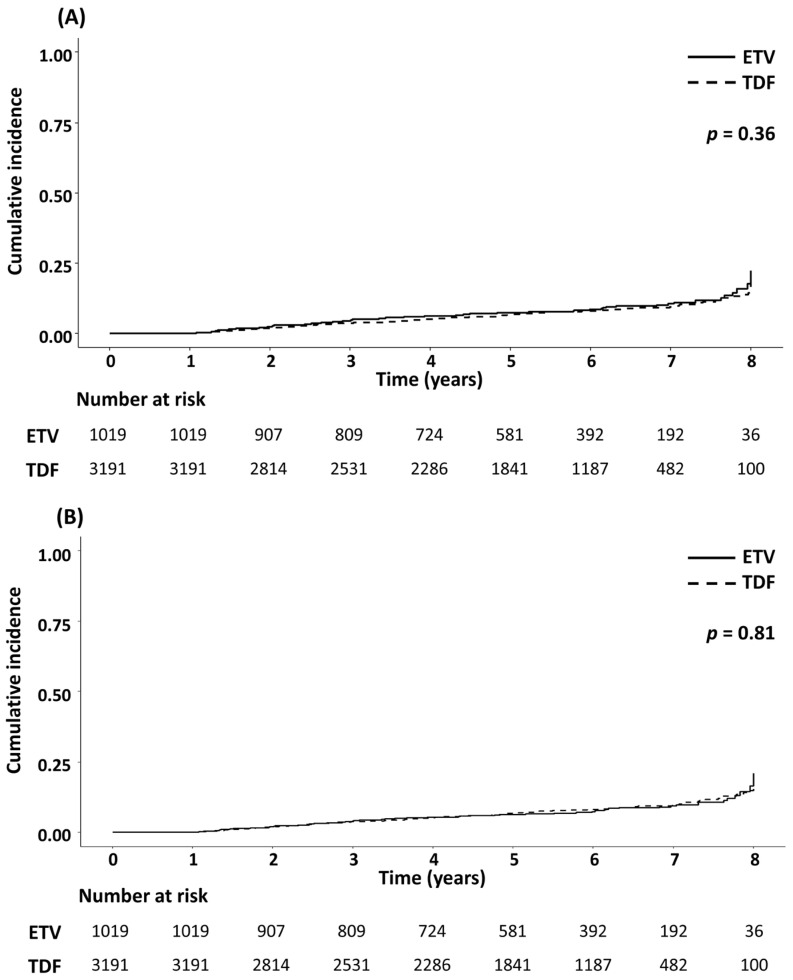
Cumulative incidence of hepatocellular carcinoma in patients treated with ETV or TDF. The Kaplan–Meier curves of ETV- or TDF-treated patients were compared in the (**A**) crude and the (**B**) IPTW-adjusted populations. ETV, entecavir; IPTW, inverse probability of treatment weighting; TDF, tenofovir disoproxil fumarate.

During a median follow-up of 5.6 and 5.5 years, 86 and 232 patients were diagnosed with HCC in the ETV and TDF groups, respectively. There was no significant difference in HCC incidence between the two groups in the crude population and the IPTW-adjusted population (both *p* > 0.05; Figure 2). Comparable results were maintained in subgroup analyses according to the HBeAg and cirrhosis status (Supplementary Figures S1 and S2). The results of univariable and multivariable Cox analyses to identify risk factors for HCC development are presented in Table 2. Older age, male sex, presence of cirrhosis, lower platelet count, serum albumin level, and HBV DNA level were significantly associated with a higher risk of HCC. However, TDF was not associated with an increased risk of HCC compared to ETV in both crude (TDF vs. ETV: adjusted HR (aHR) = 1.20, 95% CI = 0.92–1.57, *p* = 0.18) and weighted cohorts (aHR = 1.10, 95% CI = 0.84–1.42, *p* = 0.49).

### 3.3. Secondary Outcomes

During follow-up, 87 cases of mortality or LT were confirmed: 22 cases (11 deaths and 11 LT) occurred in the ETV group, and 65 cases (48 deaths and 17 LT) occurred in the TDF group. There were no significant differences in death from any cause or LT and LRO (Supplementary Figures S3 and S4). In terms of EHM, 26 and 45 patients were diagnosed with non-liver cancers in the ETV and TDF groups, respectively. The list of specific EHMs developed in both groups is summarized in Supplementary Table S1. Although the incidence of EHM was significantly higher in the ETV group than in the TDF group before weighting (*p* = 0.02), no difference was identified after IPTW was applied (*p* = 0.29; Supplementary Figure S5).

Supplementary Figure S6 depicts the incidence of newly diagnosed cirrhosis among subjects initially classified as non-cirrhotic. In the unweighted cohort, 18 (3.1%) patients in the ETV group (n = 587) and 44 (2.3%) patients in the TDF group (n = 1951) developed liver cirrhosis. The two groups demonstrated a comparable risk of cirrhosis development both before (*p* = 0.24) and after IPTW was applied (*p* = 0.39). Newly confirmed decompensation events in patients with compensated cirrhosis were also comparable between the two drugs (Supplementary Figure S7).

The results of the remaining secondary outcomes are summarized in Table 3. More than 95% of patients in each group showed CVR with no significant difference across the groups. HBeAg negative conversion rate of the ETV group (41.6%) was not significantly different from that of the TDF group (37.2%) after IPTW was applied. A total of 95 patients (32 in the ETV group and 63 in the TDF group) achieved HBsAg negative conversion. Although the proportion of seroconversion was higher in the ETV group compared to the TDF group before weighting (*p* = 0.04), no significant difference was confirmed in the balanced cohort (*p* = 0.10).

Twenty-four patients (2.4%) in the ETV group and 79 patients (2.5%) in the TDF group changed their initial antivirals during follow-up, for different reasons. Partial virologic response (45.8%) and drug side effects (82.3%) were the main causes of drug modification in the ETV and TDF groups, respectively. Sixty-five patients in the TDF group experienced drug side effects: decreased kidney function (n = 17), hypophosphatemia (n = 20), and osteoporosis (n = 18) were frequently observed. As a result, the mean adherence rate of the ETV group (0.94) was measured higher than that of the TDF group (0.92).

## 4. Discussion

In this large-scale multicenter study involving 4210 patients with CHB, we compared ETV with TDF across a wide spectrum of outcomes. During similar follow-up periods, the two antivirals were comparable regarding HCC development as well as mortality, incidence of LRO, EHM, cirrhosis development or new decompensation events, CVR, and HBeAg or HBsAg negative conversion. The TDF group experienced more side effects, leading to a lower adherence rate, compared to the ETV group.

This study has strengths in that a wide range of clinically relevant outcomes were evaluated in a large-scale cohort with similar follow-up durations. The findings of the current study can provide comprehensive information on the two most commonly used antivirals in patients with CHB. Various aspects other than HCC risk should be assessed to initiate NA treatments, especially in patients with a minimal to low risk of HCC. Since they are far less likely to develop HCC than those belonging to a high-risk group and may take antiviral drugs for the rest of their lives, several other factors may be prioritized. Similar to previous studies [26,27], patients in the TDF group experienced more side effects including renal function impairment and osteoporosis than those in the ETV group. Therefore, CHB patients who have a low risk of HCC (e.g., young, female, and non-cirrhotic patients) and marginal kidney function and/or osteopenia may benefit more from ETV than TDF. However, the incidence of drug resistance, viral breakthrough, and partial virologic response was significantly higher in the ETV group. TDF is believed to have a higher genetic barrier to resistance than ETV, as the accumulation of at least four mutations is required for the development of clinical resistance to TDF [32]. In addition, in line with current findings, previous studies showed that TDF suppressed viral RNA, as well as DNA, more effectively than ETV [6,33,34]. Therefore, TDF may be a better option for CHB patients with a higher risk of decompensation due to a partial virologic response or viral breakthrough.

In previous studies, TDF was superior or at least comparable to ETV with respect to HCC; however, these findings should be interpreted with caution. The lack of randomized trials, heterogeneity in study populations, and possible residual biases limit the validity of these observational studies. As expected, the results of meta-analyses and systematic reviews were affected by the included studies, leading to conflicting conclusions [9,10,11,12,13,14]. In addition, the earlier introduction of ETV than TDF in most countries further complicates the direct comparison of these two antivirals, as the follow-up duration may significantly vary. Some researchers hypothesized that the earlier use of ETV could also have contributed to more vulnerable patients, who had awaited the advent of potent NA, being enrolled in the ETV group, resulting in the false superiority of TDF [14,15,16]. ETV and TDF have been used in Korea since 2007 and 2012, respectively. To minimize the disparity in treatment duration, the current study included CHB patients who started NA treatment after 1 December 2012, and as a result, two antivirals could be compared regarding a number of outcomes during similar median follow-ups (5.6 vs. 5.5 years). In future studies, meta-analyses using individual patient data may provide more robust results by attenuating within-study heterogeneity after propensity score matching or covariate adjustment [35].

However, resolving controversies regarding the comparative efficacy of ETV and TDF will remain challenging, since large-scale randomized controlled trials with long-term follow-up durations are practically unfeasible. Under these circumstances, an individualized approach may be the optimal solution to determine the first-line antiviral drug, between ETV and TDF, for CHB patients. In the real world, a particular subpopulation of CHB patients is more likely to benefit from one drug than the other. Therefore, rather than concluding that all patients should take TDF or that there is no difference regardless of NA choice, a personalized strategy to select the most suitable antiviral for each patient may be an ideal compromise, and machine learning technologies are expected to be the best tool in this regard. Machine learning models can be trained to detect complex relationships among a number of variables, and they are shown to outperform previous models which adopted conventional statistical methods (e.g., the Cox proportional hazard model) [22,36]. Recently, a machine learning model has been introduced to estimate the individual risk of HCC under antiviral treatment and to suggest the appropriate first-line antivirals for each CHB patient [37]. According to this model, male and cirrhotic patients with a high risk of HCC were more likely to be recommended to use TDF as an initial treatment. To train and validate this kind of machine learning model and improve its accuracy, large-scale clinical data are required, and comprehensive data on the two antivirals from this study can serve as a valuable source.

This study has a few limitations. First, the current study was conducted in a single country and is susceptible to residual biases due to its retrospective nature. The majority of Korean CHB patients are infected with genotype C2 HBV by mother-to-child transmission [38], which raises generalizability concerns. However, CHB patients with diverse subtypes exhibited similar virologic responses to antiviral treatment [39]. To overcome the biases, multiple statistical strategies including IPTW, multivariable adjustment, and subgroup analyses were applied. Second, the number of enrolled patients in each group differed significantly. Whereas the median duration of follow-up was comparable between the two groups, the TDF group included approximately 3 times as many CHB patients as the ETV group. To balance the two groups while reducing the risk of false negativity and maximally utilize the available data, IPTW was initially chosen over propensity score matching, which is more intuitive than IPTW but has the disadvantage of excluding unmatched subjects from subsequent analyses. After applying IPTW, baseline characteristics were well balanced, and most of the findings in the crude population were reproduced. Third, CHB patients treated with TAF, a recently introduced NA, were not included. As TAF was found to have a reduced risk of adverse events compared to TDF [40,41], additional research is required to compare TAF with ETV or TDF in terms of diverse outcomes.

## 5. Conclusions

In summary, ETV and TDF exhibited comparability in several aspects including the development of HCC, mortality, the incidence of LRO or EHM, the prevention of new cirrhosis development or decompensation events, CVR, and seroconversion rates after being balanced using IPTW. In the TDF group, more treatment-related side effects were reported, such as impaired renal function, hypophosphatemia, and osteoporosis. On the other hand, higher rates of drug resistance, viral breakthrough, and partial virologic response were observed in the ETV group. Further research is warranted to compare the long-term outcomes of antivirals including TAF.

## Figures and Tables

**Table 1:** Baseline characteristics of the study cohort.

	Before IPTW			After IPTW			
	ETV Group (n = 1019)	TDF Group (n = 3191)	*p* Value	ETV Group	TDF Group	*p* Value	SMD
Age, years	52 (44–60)	49 (40–57)	<0.001	50 (41–57)	50 (41–57)	0.73	0.01
Male, %	60.5	61.3	0.67	60.5	60.2	0.90	0.006
BMI, kg/m^2^	23.7 (21.7–25.9)	23.6 (21.5–25.8)	0.52	23.4 (21.0–25.6)	23.2 (20.8–25.5)	0.36	0.02
Diabetes mellitus, %	11.3	8.0	0.002	8.9	8.6	0.82	0.008
Hypertension, %	13.8	7.8	<0.001	9.2	8.9	0.73	0.01
Cirrhosis, %	42.4	38.9	0.049	39.6	38.9	0.73	0.01
Decompensated cirrhosis, %	5.7	5.5	0.83	5.3	5.4	0.98	0.001
HBeAg positive, %	41.9	50.3	<0.001	48.3	47.5	0.71	0.02
Platelet count, ×1000/mm^3^	157 (115–200)	164 (122–205)	0.005	161 (120–207)	161 (121–203)	0.85	0.02
Albumin, g/dL	4.2 (3.8–4.4)	4.2 (3.9–4.4)	0.19	4.2 (3.8–4.4)	4.2 (3.8–4.4)	0.68	0.03
Bilirubin, mg/dL	0.9 (0.6–1.3)	0.9 (0.6–1.2)	0.99	0.9 (0.7–1.3)	0.9 (0.7–1.2)	0.96	0.002
AST, U/L	64 (41–117)	68 (43–120)	0.003	67 (41–122)	68 (43–124)	0.17	0.06
ALT, U/L	80 (41–156)	90 (48–172)	<0.001	86 (44–176)	89 (48–178)	0.16	0.05
Prothrombin time, INR	1.1 (1.0–1.1)	1.1 (1.0–1.1)	0.65	1.1 (1.0–1.1)	1.1 (1.0–1.1)	0.89	0.005
Creatinine, mg/dL	0.8 (0.7–1.0)	0.8 (0.7–0.9)	<0.001	0.8 (0.7–1.0)	0.8 (0.7–1.0)	0.74	0.006
AFP, ng/mL	4.3 (2.6–9.3)	4.7 (2.8–11.0)	0.02	4.4 (2.7–10.8)	4.5 (2.7–10.9)	0.63	0.003
HBV DNA, log_10_ IU/mL	6.2 (4.9–7.5)	6.4 (5.2–7.8)	0.002	6.3 (5.1–7.7)	6.4 (5.2–7.6)	0.78	0.008

Data are expressed as % or median (interquartile range). AFP, alpha fetoprotein; ALT, alanine aminotransferase; AST, aspartate aminotransferase; BMI, body mass index; ETV, entecavir; HBeAg, hepatitis B e antigen; HBV, hepatitis B virus; IPTW, inverse probability of treatment weighting; TDF, tenofovir disoproxil fumarate.

**Table 2:** Univariable and multivariable Cox analyses for hepatocellular carcinoma incidence.

Characteristics	Before IPTW				After IPTW			
	Univariable		Multivariable		Univariable		Multivariable	
	HR	*p* Value	aHR	*p* Value	HR	*p* Value	aHR	*p* Value
TDF (vs. ETV)	0.89 (0.69–1.14)	0.35	1.20 (0.92–1.57)	0.18	1.03 (0.80–1.33)	0.83	1.10 (0.84–1.42)	0.49
Age	1.05 (1.04–1.06)	<0.001	1.04 (1.03–1.05)	<0.001	1.05 (1.04–1.07)	<0.001	1.04 (1.03–1.05)	<0.001
Male (vs. Female)	1.61 (1.26–2.05)	<0.001	2.03 (1.55–2.65)	<0.001	1.41 (1.06–1.85)	0.02	1.92 (1.43–2.63)	<0.001
BMI, kg/m^2^	1.02 (0.98–1.05)	0.37			1.04 (1.01–1.07)	0.03	1.02 (0.99–1.06)	0.19
Diabetes mellitus	1.86 (1.37–2.52)	<0.001	1.21 (0.88–1.68)	0.24	1.76 (1.24–2.49)	0.001	1.11 (0.77–1.61)	0.58
Hypertension	1.79 (1.33–2.41)	<0.001	1.26 (0.91–1.74)	0.17	1.70 (1.23–2.35)	0.001	1.14 (0.79–1.66)	0.48
Cirrhosis	4.07 (3.18–5.21)	<0.001	1.99 (1.49–2.66)	<0.001	3.79 (2.86–5.03)	<0.001	1.89 (1.36–2.62)	<0.001
HBeAg positive	0.74 (0.58–0.91)	0.005	1.11 (0.85–1.44)	0.44	0.68 (0.53–0.89)	0.005	1.04 (0.76–1.43)	0.81
Platelet count, ×1000/mm^3^	0.99 (0.98–0.99)	<0.001	0.99 (0.98–0.99)	<0.001	0.99 (0.98–0.99)	<0.001	0.99 (0.98–0.99)	<0.001
Albumin, g/dL	0.40 (0.34–0.47)	<0.001	0.69 (0.55–0.88)	0.002	0.43 (0.36–0.51)	<0.001	0.60 (0.46–0.77)	<0.001
Total bilirubin, mg/dL	1.03 (0.99–1.08)	0.14			1.03 (1.01–1.06)	0.02	0.94 (0.88–1.01)	0.052
ALT, U/L	1.00 (0.99–1.00)	0.10			1.00 (0.99–1.00)	0.12		
Prothrombin time, INR	1.88 (1.58–2.22)	<0.001	1.23 (0.69–2.22)	0.48	1.97 (1.61–2.41)	<0.001	1.30 (0.71–2.35)	0.40
HBV DNA, log_10_ IU/mL	0.83 (0.77–0.89)	<0.001	0.90 (0.83–0.99)	0.02	0.82 (0.76–0.89)	<0.001	0.89 (0.81–0.98)	0.02

Data are expressed as hazard ratio (95% confidence interval). aHR, adjusted hazard ratio; ALT, alanine aminotransferase; BMI, body mass index; ETV, entecavir; HBeAg, hepatitis B e antigen; HBV, hepatitis B virus; HR, hazard ratio; IPTW, inverse probability of treatment weighting; TDF, tenofovir disoproxil fumarate.

**Table 3:** Comparison of the two antivirals in terms of virologic response, HBeAg or HBsAg negative conversion, adherence rate, and drug modification rate.

	Before IPTW			After IPTW		
	ETV Group (n = 1019)	TDF Group (n = 3191)	*p* Value	ETV Group	TDF Group	*p* Value
Complete virologic response, %	95.8	95.6	0.80	95.1	95.8	0.38
HBeAg negative conversion, %	41.1	38.4	0.25	41.6	37.2	0.09
HBsAg negative conversion, %	3.1	2.0	0.04	2.8	1.9	0.10
Mean adherence rate	0.94 ± 0.16	0.92 ± 0.16	<0.001	0.94 ± 0.15	0.92 ± 0.15	0.01
Drug modification, %	2.4	2.5	0.92	2.6	2.5	0.89
Reason for drug modification (n = 103)	ETV group (n = 24)	TDF group (n = 79)	*p* value	ETV group	TDF group	*p* value
			<0.001			<0.001
Drug resistance, %	12.5	2.5		13.9	2.2	
Viral breakthrough, %	16.7	3.8		14.0	3.6	
Partial virologic response, %	45.8	6.3		45.7	7.0	
Side effects, %	8.3	82.3		8.5	82.5	
Other, %	16.7	5.1		18.0	4.7	

Data are expressed as % or mean ± standard deviation. ETV, entecavir; HBeAg, hepatitis B e antigen; HBsAg, hepatitis B s antigen; IPTW, inverse probability of treatment weighting; TDF, tenofovir disoproxil fumarate.

## Data Availability

The data presented in this study are available on request from the corresponding author. The data are not publicly available due to privacy.

## Supplementary Materials

The following supporting information can be downloaded at https://www.mdpi.com/article/10.3390/cancers15112936/s1: Table S1: The list of extrahepatic malignancies diagnosed in the ETV and TDF groups during follow-up; Figure S1: Cumulative incidence of hepatocellular carcinoma according to the HBeAg positivity; Figure S2: Cumulative incidence of hepatocellular carcinoma according to the presence of liver cirrhosis; Figure S3: Cumulative incidence of death or liver transplantation in patients treated with ETV or TDF; Figure S4: Cumulative incidence of liver-related outcome, defined as hepatocellular carcinoma, liver transplantation, or liver-related death; Figure S5: Cumulative incidence of overall extrahepatic malignancy: Figure S6: Cumulative incidence of newly diagnosed liver cirrhosis in initially non-cirrhotic patients; Figure S7: Cumulative incidence of new decompensated events (variceal bleeding, hepatic encephalopathy, or ascites) in initially compensated liver cirrhosis patients.
