# Supplementary material for: Inverse Propensity Score-Weighted Analysis of Entecavir and Tenofovir Disoproxil Fumarate in Patients with Chronic Hepatitis B: A Large-Scale Multicenter Study
Source: Cancers (Basel). 2023 May 26;15(11):2936. doi: 10.3390/cancers15112936 (PMC10252077; doi:10.3390/cancers15112936)
Supplement: Supplementary file 1 [file cancers-15-02936-s001.zip › cancers-2355344-supplementary.pdf]

Supplementary Material: Inverse Propensity Score-Weighted Analysis of Entecavir and Tenofovir Disoproxil Fumarate in Patients with Chronic Hepatitis B: A Large-Scale Multicenter Study

Jihye Kim, Moon Haeng Hur, Seung Up Kim, Jin-Wook Kim, Dong Hyun Sinn, Hyun Woong Lee, Moon Young Kim, Jae Youn Cheong, Yong Jin Jung, Han Ah Lee, Young-Joo Jin, Jun Sik Yoon, Sung-Jae Park, Chang Hun Lee, In Hee Kim, June Sung Lee, Young Youn Cho, Hyung Joon Kim, Soo Young Park, Yeon Seok Seo, Hyunwoo Oh, Dae Won Jun, Mi Na Kim, Young Chang, Jae Young Jang, Sang Youn Hwang and Yoon Jun Kim*

Table S1. The list of extrahepatic malignancy diagnosed in the ETV and TDF groups during follow-up. ETV, entecavir; TDF, tenofovir disoproxil fumarate.

	ETV group, n	TDF group, n
Gastric cancer	5	5
Colon cancer	1	5
Gallbladder and biliary tract cancer	2	5
Thyroid cancer	3	3
Lung cancer	3	0
Breast cancer	1	4
Lymphoma	3	2
Prostate cancer	2	0
Renal pelvis cancer	1	0
Pancreatic cancer	0	1
Cervical cancer	0	1
Ovarian cancer	0	1
Brain cancer	0	1
Leukemia	0	1
Melanoma	0	1
Orbital cancer	0	1
Parotid gland cancer	0	1
Tonsillar cancer	0	1
Vocal fold cancer	0	1
Others	1	1
Unknown	4	10
**Total**	26	45

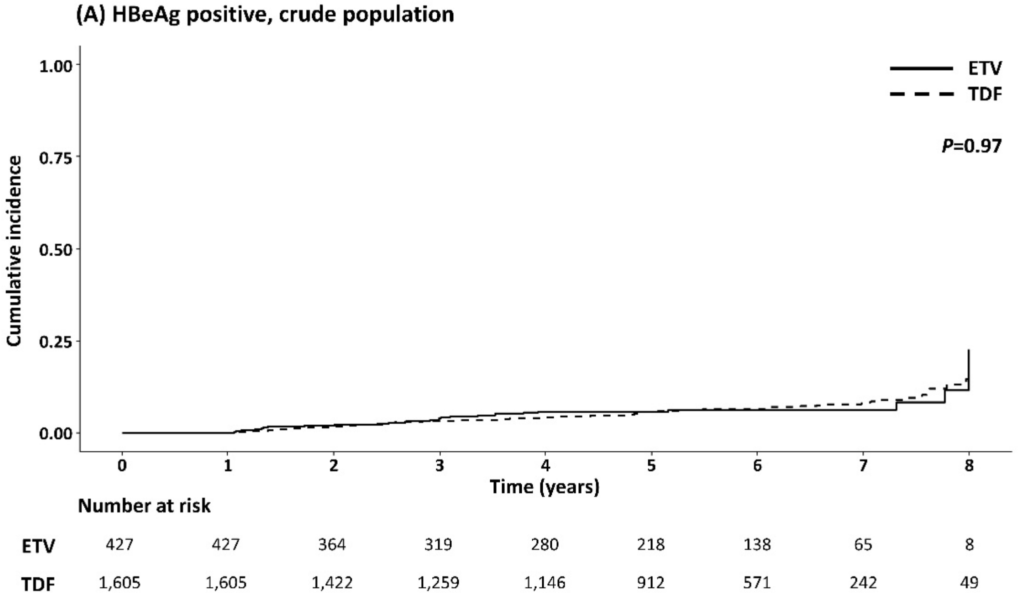

(B) HBeAg positive, after IPTW

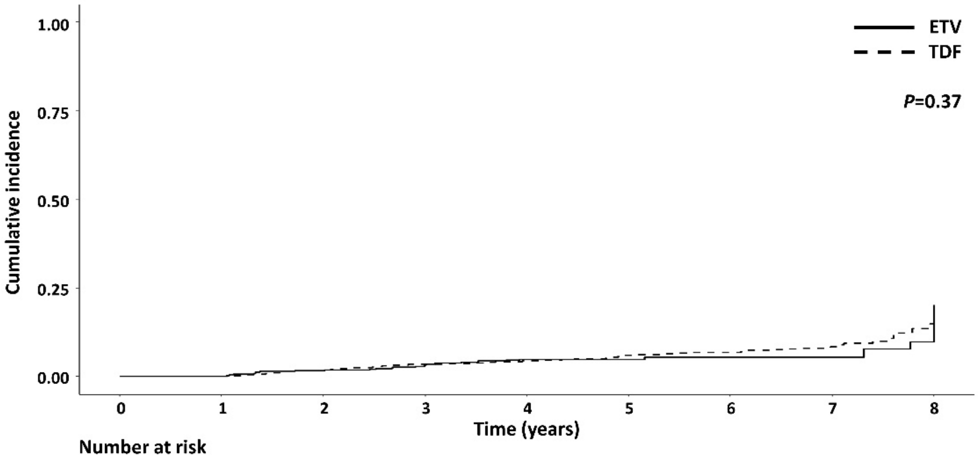

Number at risk

**ETV**	427	427	364	319	280	218	138	65	8
**TDF**	1,605	1,605	1,422	1,259	1,146	912	571	242	49

(C) HBeAg negative, crude population

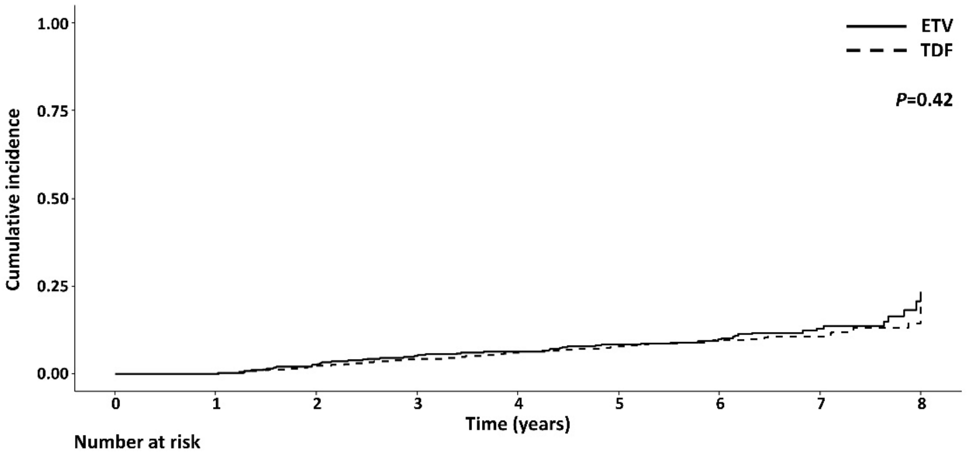

Number at risk

**ETV**	592	592	543	490	444	363	254	127	28
**TDF**	1,586	1,586	1,392	1,272	1,140	929	616	240	51

(D) HBeAg negative, after IPTW

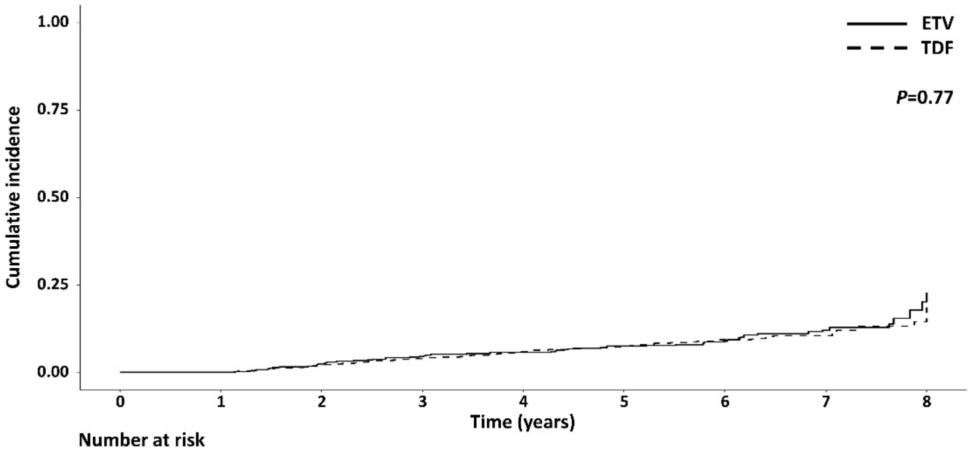

Number at risk

**ETV**	592	592	543	490	444	363	254	127	28
**TDF**	1,586	1,586	1,392	1,272	1,140	929	616	240	51

Figure S1. Cumulative incidence of hepatocellular carcinoma according to the HBeAg positivity. The Kaplan-Meier curves of ETV- or TDF-treated patients with positive HBeAg were compared in

the (A) crude and the (B) IPTW-adjusted populations. Same analyses were performed in patients with negative HBeAg (C) before and (D) after applying IPTW. ETV, entecavir; HBeAg, hepatitis B e antigen; IPTW, inverse probability of treatment weighting; TDF, tenofovir disoproxil fumarate.

(A) With liver cirrhosis, crude population

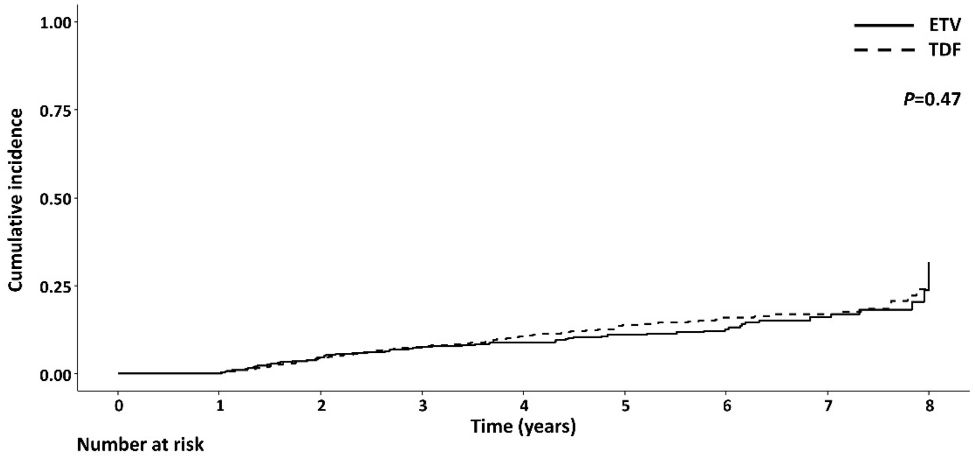

Number at risk

**ETV**	432	432	382	357	331	266	189	90	19
**TDF**	1,240	1,240	1,101	989	880	700	444	164	33

(B) With liver cirrhosis, after IPTW

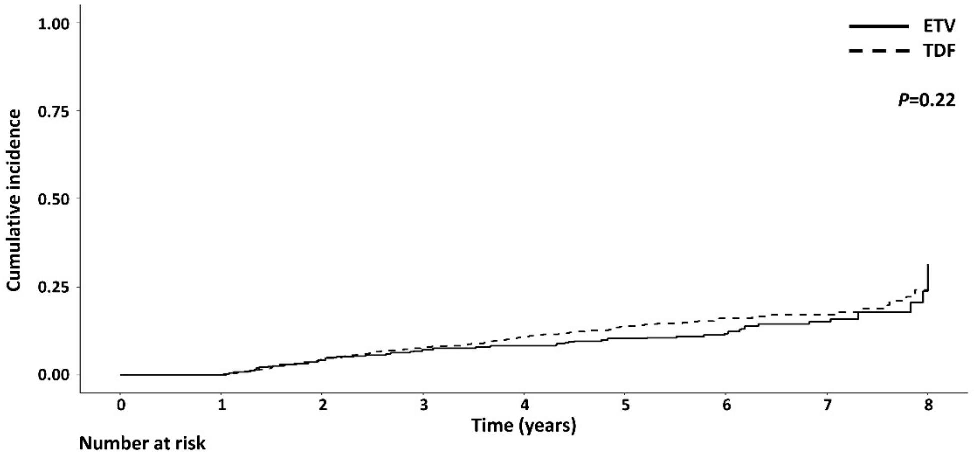

Number at risk

**ETV**	432	432	382	357	331	266	189	90	19
**TDF**	1,240	1,240	1,101	989	880	700	444	164	33

(C) Without liver cirrhosis, crude population

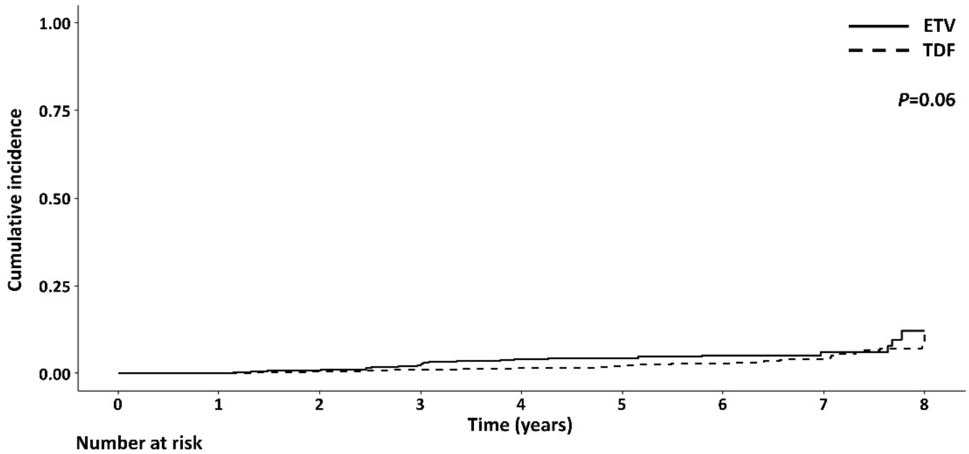

Number at risk

**ETV**	587	587	525	452	393	315	203	102	17
**TDF**	1,951	1,951	1,713	1,542	1,406	1,141	743	318	67

(D) Without liver cirrhosis, after IPTW

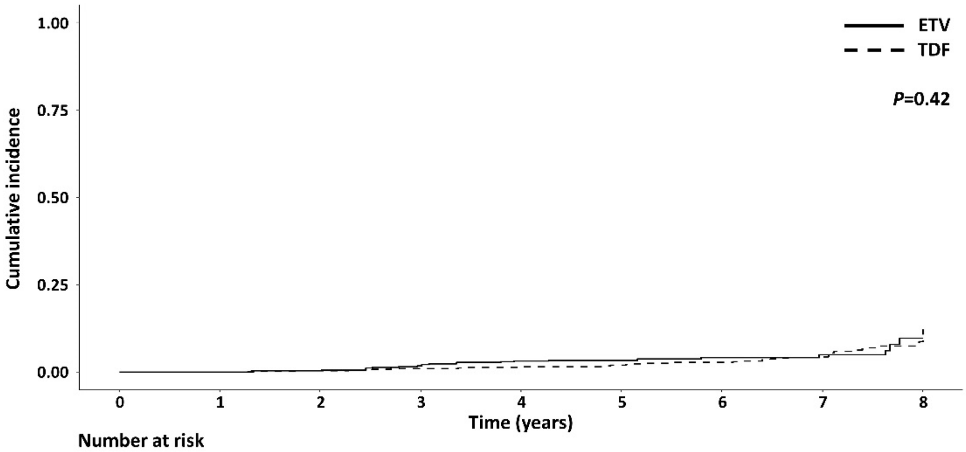

Number at risk

**ETV**	587	587	525	452	393	315	203	102	17
**TDF**	1,951	1,951	1,713	1,542	1,406	1,141	743	318	67

Figure S2. Cumulative incidence of hepatocellular carcinoma according to the presence of liver cirrhosis. The Kaplan-Meier curves of ETV- or TDF-treated patients with liver cirrhosis were compared in the (A) crude and the (B) IPTW-adjusted populations. Same analyses were performed in patients without liver cirrhosis (C) before and (D) after applying IPTW. ETV, entecavir; IPTW, inverse probability of treatment weighting; TDF, tenofovir disoproxil fumarate.

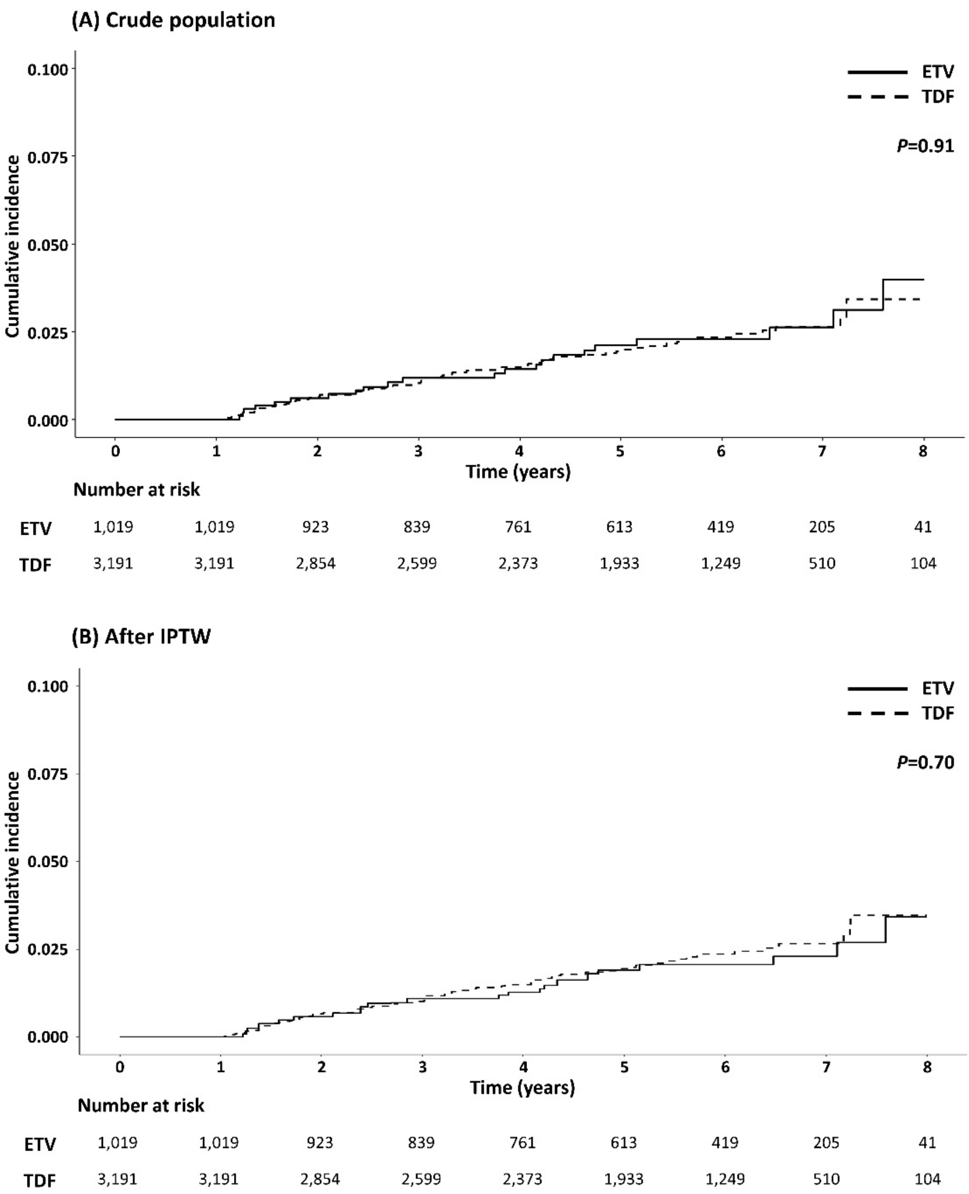

Figure S3. Cumulative incidence of death or liver transplantation in patients treated with ETV or TDF. Patients treated with either ETV or TDF were compared in the (A) crude and the (B) IPTW-adjusted populations. ETV, entecavir; IPTW, inverse probability of treatment weighting; TDF, tenofovir disoproxil fumarate.

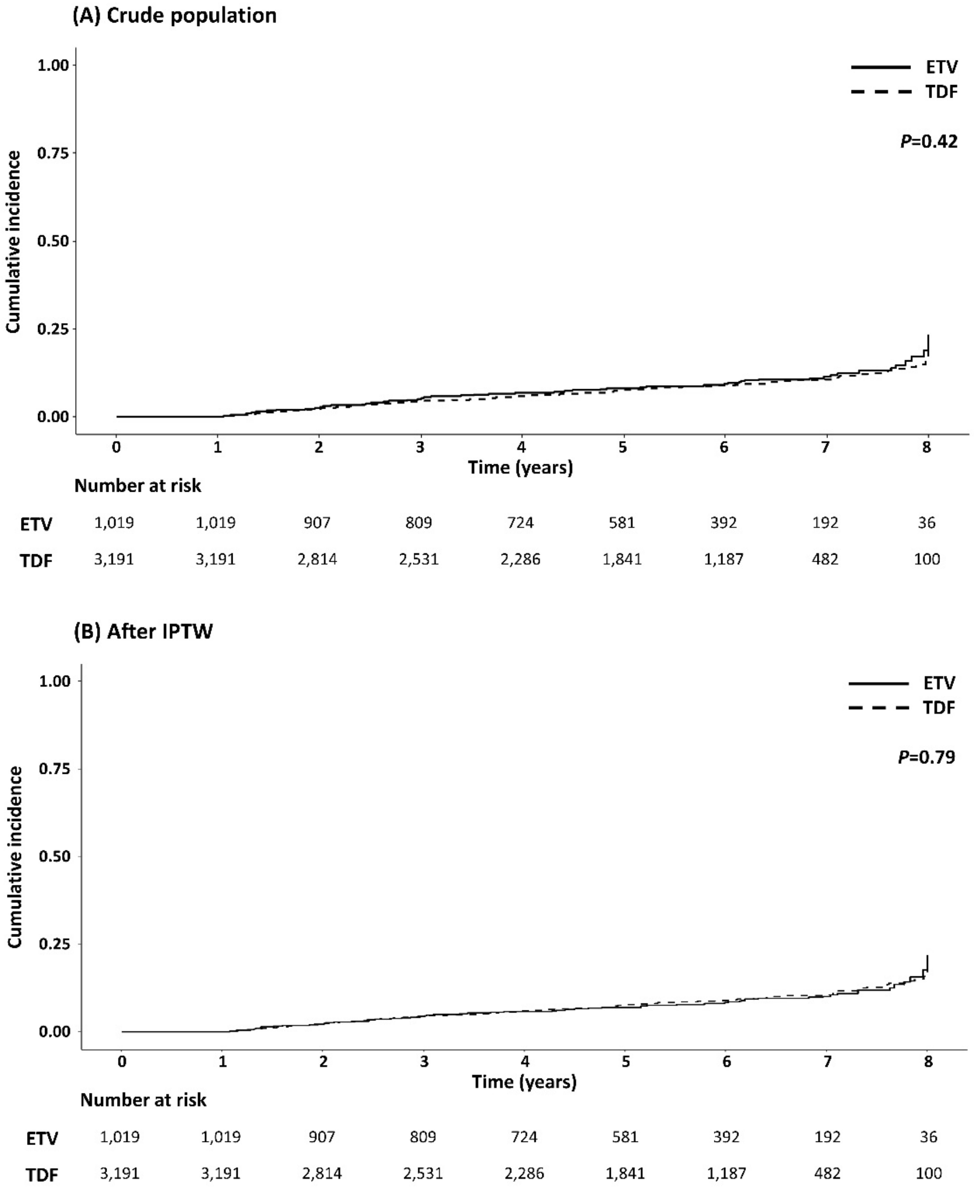

Figure S4. Cumulative incidence of liver-related outcome, defined as hepatocellular carcinoma, liver transplantation, or liver-related death. Patients treated with either ETV or TDF were compared in the (A) crude and the (B) IPTW-adjusted populations. ETV, entecavir; IPTW, inverse probability of treatment weighting; TDF, tenofovir disoproxil fumarate.

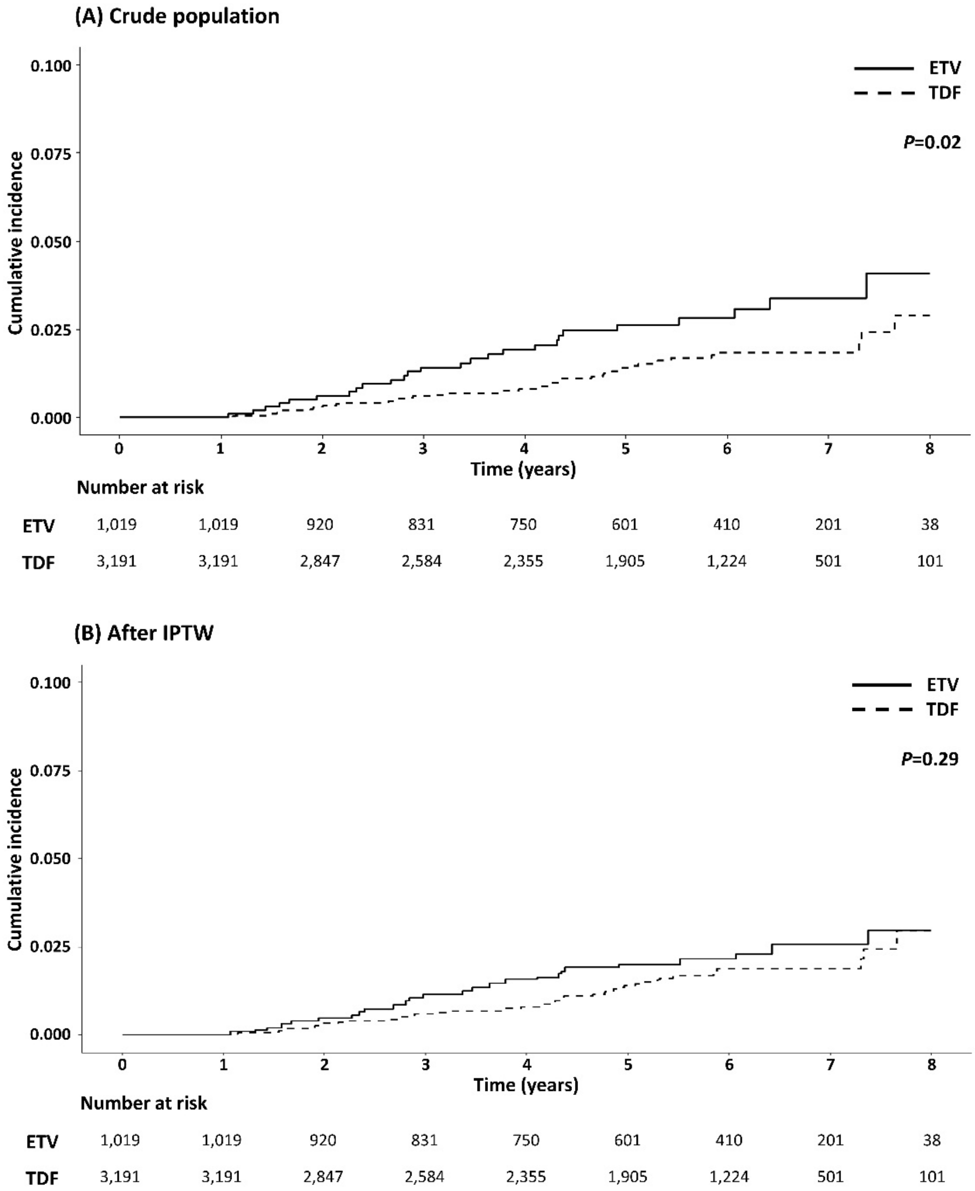

Figure S5. Cumulative incidence of overall extrahepatic malignancy. Patients treated with either ETV or TDF were compared in the (A) crude and the (B) IPTW-adjusted populations. ETV, entecavir; IPTW, inverse probability of treatment weighting; TDF, tenofovir disoproxil fumarate.

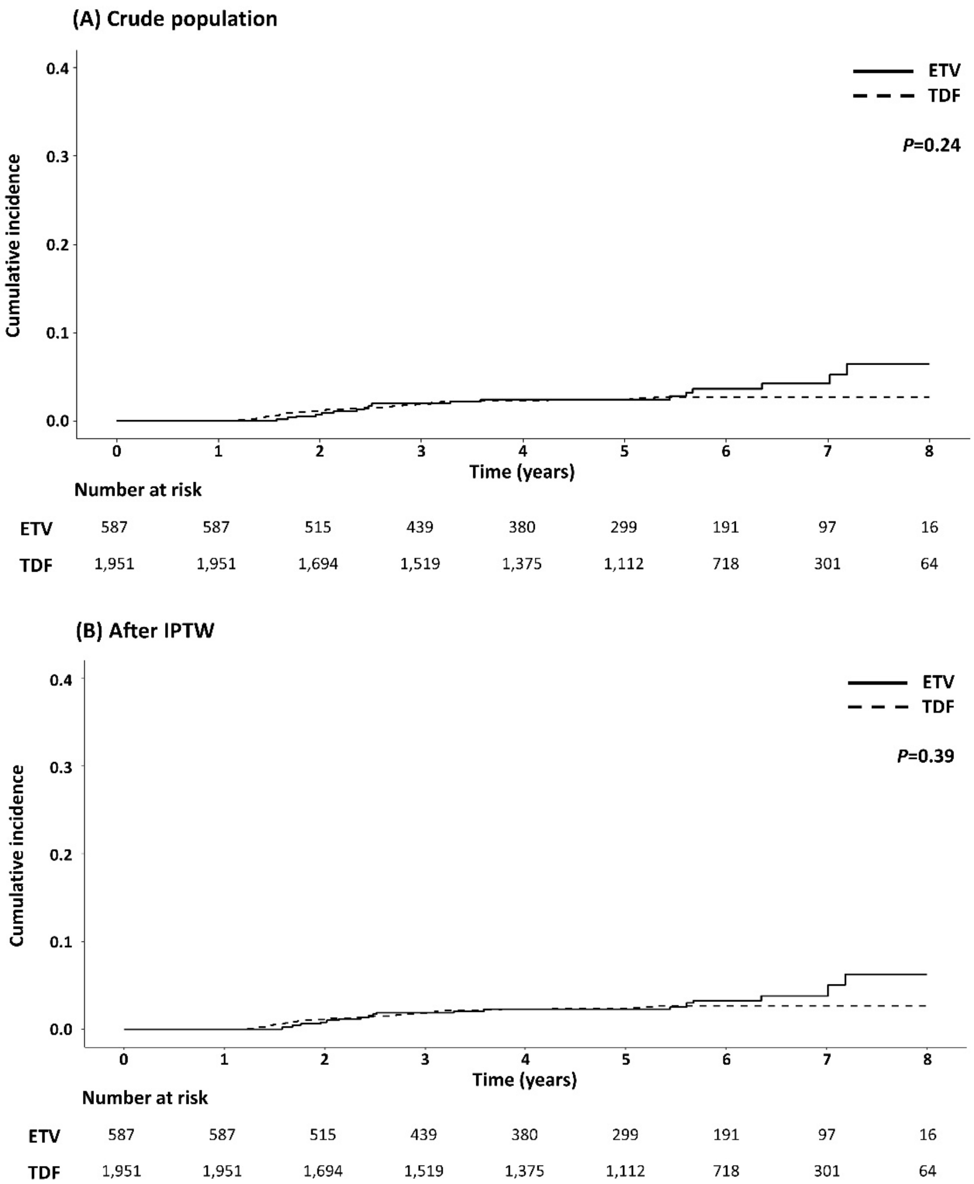

Figure S6. Cumulative incidence of newly diagnosed liver cirrhosis in initially non-cirrhotic patients. The Kaplan-Meier curves of ETV- or TDF-treated patients were compared in the (A) crude and the (B) IPTW-adjusted populations. ETV, entecavir; IPTW, inverse probability of treatment weighting; TDF, tenofovir disoproxil fumarate.

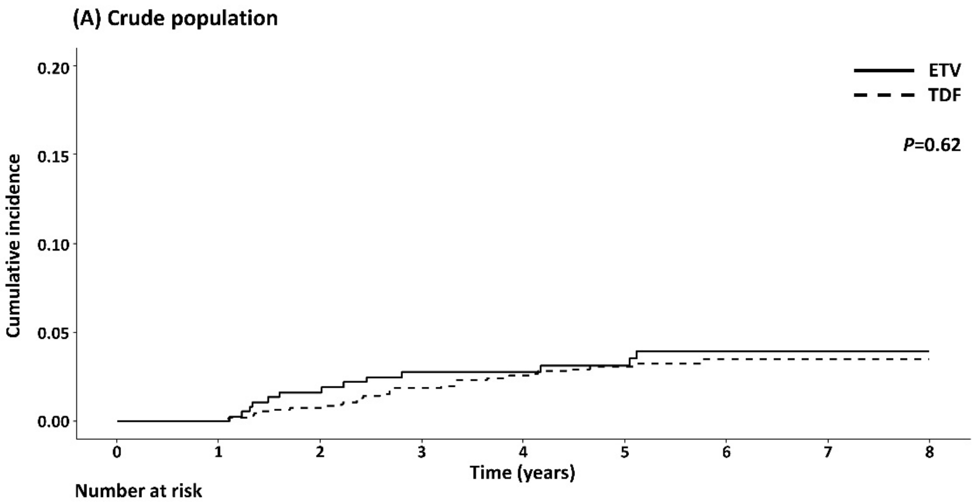

Number at risk

	0	1	2	3	4	5	6	7	8
**ETV**	378	378	344	319	299	240	172	86	19
**TDF**	1,078	1,078	966	870	778	621	397	144	31

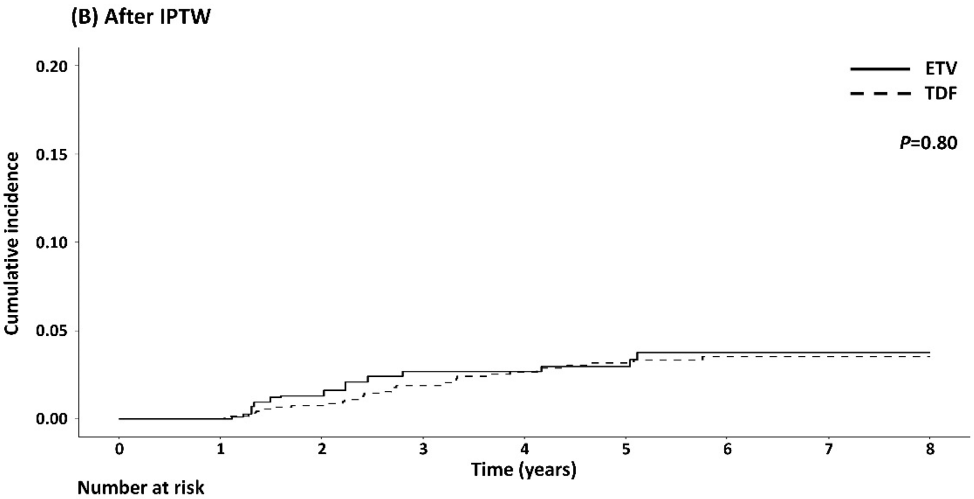

Number at risk

	0	1	2	3	4	5	6	7	8
**ETV**	378	378	344	319	299	240	172	86	19
**TDF**	1,078	1,078	966	870	778	621	397	144	31

Figure S7. Cumulative incidence of new decompensated events (variceal bleeding, hepatic encephalopathy, or ascites) in initially compensated liver cirrhosis patients. Patients treated with either ETV or TDF were compared in the (A) crude and the (B) IPTW-adjusted populations. ETV, entecavir; IPTW, inverse probability of treatment weighting; TDF, tenofovir disoproxil fumarate.
